# Mapping the research landscape of mHealth and technology in pediatric chronic illness: a bibliometric study

**DOI:** 10.3389/fdgth.2025.1540362

**Published:** 2025-03-31

**Authors:** Esther Rodríguez-Jiménez, Javier Martín-Ávila, Selene Valero-Moreno, Marián Pérez-Marín

**Affiliations:** Departamento de Personalidad, Evaluación y Tratamientos Psicológicos, Facultad de Psicología y Logopedia, Universitat de València, Valencia, Spain

**Keywords:** chronic disease, adolescents, pediatric, mHealth, digital intervention, new technologies, self-management, apps

## Abstract

**Introduction:**

The presence of a chronic disease during adolescence has been linked to an increased risk of developing psychosocial problems and a greater likelihood of experiencing difficulties during the transition to adulthood. In this regard, research has been conducted on the development of applications or programs based on new technologies to address the potential complications associated with self-management and coping with chronic diseases in adolescents.

**Method:**

The objective of the present study was to conduct a bibliometric analysis of the existing literature on the use of new technologies to enhance self-management and coping with chronic diseases during adolescence. This four-staged bibliometric analysis was conducted using the following software programs: HisCite, Bibexcel, Pajek, and VOSviewer. The programs were utilized for the extraction of data and the subsequent construction of graphs, which facilitate the visualization of existing networks between disparate authors, institutions, and terms.

**Results:**

The screening results shortlisted a total of 157 articles from an initial 207. The further analysis of these records indicated that the United States was the most prominent nation in terms of scientific output in the domain of digital applications designed to enhance self-management and coping in chronic diseases during adolescence.

**Discussion:**

The digital intervention in this specific population is primarily associated with the development of the mHealth app, along with the physical and psychological consequences that illness can entail. This research can serve as a reference for future bibliometric studies or scientific investigations in this field.

## 1 Introduction

Chronic diseases (CD) are the leading cause of death worldwide, accounting for a high percentage of human losses (74%). Among these, the most prevalent are cardiovascular diseases, cancer, respiratory diseases and diabetes (1). The definition of CD is complicated by the variety of particularities each of they may present, but they can be conceptualized as long-term conditions that currently have no cure, that impact on different areas of daily life, and whose treatment focuses on controlling and even ameliorating symptoms, as well as facilitating the social adaptation of sufferers (2–6).

The present situation about these diseases is one of increasing prevalence, especially among the pediatric and adolescent population (Brady et al., 2021; Kennedy et al., 2024) (7, 8). Although the exact number of children and adolescents with CD is unknown to date (9), some studies estimate that one in four children suffers from CD (6). Advances in the diagnosis and treatment of these conditions have resulted in improved life expectancy for these patients (10, 11), which, in turn, has led to increased rates of psychological disorders in the pediatric population (9, 12, 13). In adolescence, a few age-related biopsychosocial changes occur, which may be influenced by the presence of CD, impacting on their quality of life (14–16). Furthermore, these adolescents encounter various challenges associated with their condition, including hospitalization and the development of secondary symptoms—such as pain or fatigue–, physical ailments and emotional distress (11). Due to its chronic nature, these conditions have been shown to have significant psychosocial consequences for the individual and their family environment (17). Living with CD involves a strict regimen of medical follow-up and treatment guidelines, which often place an added burden of stress on sufferers and affect social relationships and the performance of daily activities (18–20). During childhood, the primary caregivers are responsible for the management of the disease (21, 22) However, over the years and with the transition to adolescence—characterized mainly as a time of physical and emotional maturation, identity formation and peer group importance (11, 23, 24)–, these adolescents tend to exhibit an increased level of autonomy and independence, often assuming a greater degree of responsibility for their own self-care in relation to their illness (25). In this context, the development of self-management skills is of paramount importance in assisting adolescents to effectively manage and cope with the disease and its associated consequences (26). A substantial body of research has examined the psychological impact of these diseases, frequently identifying a high comorbidity with mental disorders such as depression, schizophrenia, anxiety disorders or bipolar disorder (27), which underscores the interconnection between physical and mental health, with individuals diagnosed with CD being more prone to developing psychological disorders and vice versa (9, 28). In general, individuals with CD face a heightened risk of internalizing and externalizing problems compared to those without the condition (29). The prevalence of anxious and depressive symptomatology in pediatric CD populations has been extensively documented in various studies (30), with those with CD demonstrating a higher propensity to develop psychological problems compared to those without the condition (9). Adolescents with the condition have been shown to experience a greater social impact and lower quality of life and physical well-being in comparison to their peers without the disease (13, 31, 32). Extant literature documents a general decline in the various domains of life among these adolescents, who report a perceived diminution in social support and a higher prevalence of emotional and behavioral difficulties compared to their peers without the disease (11). Furthermore, adherence to treatment is a pivotal factor given its impact on the quality of life and the physical and psychological health of the individual (30).

As a higher number of exacerbations or disease-related problems are often observed during this transition from one developmental stage to another (26), it is important to develop effective interventions that are accessible and appealing to these ages, and that improve and promote adherence to treatment and self-management patterns. Furthermore, if these problems are not adequately addressed, they may have a negative impact on development into adulthood (33). Addressing the needs of these patients on an ongoing basis outside the hospital setting can become a difficult task, and they often do not receive adequate care and attention, lacking the facilities to make a successful transition to adulthood (34–36).

To surmount this obstacle and facilitate access to this population, technological advances have enabled the development of digital applications with specific objectives focused on coping, self-management and the prevention of chronic diseases (37, 38). Among the broad spectrum of these applications, mHealth has emerged as a prominent, readily accessible, and cost-effective option (39), facilitating the monitoring of various dimensions associated with the disease (40). Returning to the genesis of the concept, the inaugural publication focusing predominantly on mHealth systems was published in 2006. In this seminal paper, the concept was defined as “emerging mobile communications and network technologies for healthcare”, signifying an evolution from the typically static telecare format to a mobile and more flexible one, with the possibility of also being used on smartphones and tablets (41).

Conversely, serious games represent a digital format that has seen increased utilization for the purpose of intervention in diverse populations and conditions. These games, or activities, are characterized by their overarching objective of achieving a higher purpose than that of the game itself. In this manner, they facilitate the acquisition of a range of skills by the user, employing a format that is both enjoyable and engaging (42). Due to their interactive nature, these procedures have been extensively utilized as intervention and learning methods in various contexts, enhancing the motivation and involvement of participants (43). The integration of these programs or application types, in conjunction with AI-powered mHealth, facilitates real-time, personalized, non-invasive care and attention, offering a non-intrusive approach (44).

In the domain of CD, and during the adolescent period, this type of intervention has been demonstrated to be efficacious in facilitating meaningful learning and self-management skills, with a positive impact on adjustment to the disease (45). A plethora of digital interventions in various formats are currently available, focusing on various chronic diseases such as diabetes (46–48), asthma (49) or cystic fibrosis (50, 51). Despite the development of numerous applications targeting this demographic over the years, a significant proportion of these applications prioritize medical aspects, often neglecting to address the psychosocial dimension.

In consideration of the points, the objective of the present paper is to offer, by means of a bibliometric analysis, a comprehensive and contemporary perspective on the extant studies published to date concerning the development of digital applications with a focus on self-management and coping with chronic diseases in the adolescent population. Bibliometric analyses are precisely useful for visualizing the scientific literature published to date on a specific topic, allowing researchers to extract, map and make sense of large volumes of quantitative data on the accumulated knowledge in an area of study and its nuances (52, 53).

Given the dearth of research in this field, the main contribution of the present study is to address a narrow area of inquiry that has not yet been subjected to bibliometric analysis. Therefore, the principal objective here is to conduct a comprehensive examination of the current state of research on the development of digital applications for self-management and coping with chronic diseases during adolescence. To this end, an analysis was conducted of scientific articles published in the *Web of Science* (WoS) database on any technological or digital application or program focused on aspects related to self-management and coping with chronic diseases in adolescents. The research questions posed were as follows: (1) At what point did this line of research emerge and how did it evolve within the catalog of articles in Web of Science? (2) Which authors and institutions are most relevant in relation to this topic; and, finally, (3) Which are the main journals specializing in this area of study?

## 2 Materials and methods

As previously stated, the objective of the present study is to conduct a bibliometric analysis of the most significant articles published to date on the application of new technologies in adolescents with chronic diseases. Such an analysis enables the quantification of disciplinary contributions, detection of emerging themes, and evaluation of collaborative networks within this interdisciplinary field. By synthesizing publication patterns, citation dynamics, and thematic clusters, this approach provides empirical insights into how digital tools are being studied and implemented to address chronic disease management. This type of study can help to systematically map the evolving research landscape, identify knowledge gaps, and inform future research prioritization and policy development (47). To this end, the number of publications, institutions, countries, and most outstanding authors, references, most cited articles, most used key terms, co-citations, and co-authorships are analyzed and presented in detail. The search and analysis were conducted in accordance with the Science Citation Index (SCI) impact factor through the *Web of Science* (WoS) Core Collection database (54). The Web of Science (WoS) database was selected as the primary data source for this bibliometric analysis since is considered the most widely accepted database for extracting and analyzing scientific articles (55).Temporal coverage and citation data integrity were additional determinants in selecting WoS, which includes publication and citation metadata dating to 1,900, enabling longitudinal analysis (56). Alternative platforms such as Google Scholar were excluded due to documented inconsistencies in citation accuracy, while PubMed lacks native citation analysis tools (57). Finally, WoS was prioritized for its superior journal classification system relative to Scopus, which enhances precision in disciplinary categorization and thematic mapping (58).

### 2.1 Design

A quantitative study was conducted to examine the literature on the relationship between new technologies or technological interventions in chronic disease during the period of adolescence, from the years up to the present.

To analyze the results, descriptive methods, descriptive bibliometric analyses, and bibliometric mappings were employed.

### 2.2 Data collection

The search was conducted using the Web of Science Core Collection database on SCI-EXPANDED and SSCI. The following equation was used to conduct the search: *[TS* *=* *(chronic illness OR chronic disease OR chronic condition)] AND TS* *=* (adolesc* OR teen*)) AND TS *=* *(mHealth OR digital interventions OR serious games OR digital technology)*. Furthermore, only those articles published in scientific journals were included, with the document type selected as either “Article” or “Review Article.” Given the limited number of publications in this field, no filtering was applied at the year level. The search yielded a total of 207 articles, of which 50 were excluded on the grounds that they were based on purely medical content. During the process of data homogenization, an additional six papers were excluded from the analysis on the grounds that they were conference publications. In conclusion, a total of 157 articles were selected for analysis in the present study.

### 2.3 Data analysis

The bibliometric analysis of the selected articles was carried out using four different programs. Firstly, *HisCite* (version 12.3.17) is a program whose aim is to facilitate the analysis and measurement of scientific and academic production by researchers, institutions, years, etc., as well as impact indicators of scientific publications and articles (59). Secondly, *Bibexcel* (updated in 2017) is a tool designed for the analysis of bibliographic data, which can be extracted in different formats and then imported and processed by other programs (60). On the other hand, *Pajek* (version 5.19) is software focused on the analysis and visualization of social networks between different authors or institutions related to each other (61). Finally, *VOSviewer* (version 1.6.20) is a software tool that allows the visual construction of bibliometric networks through citation, co-citation or co-authorship relationships between researchers or journals, as well as co-occurrence networks between keywords (62). The decision to utilize these programs was made on the basis of their extensive utilization in bibliometric studies (52, 63) complemented by their accessibility and ease of use, given their status as free and open-source software. Additionally, their compatibility with the WoS database was a determining factor in their selection.

To extract the data correctly in tabular and graphical form, the data were first cleansed through a homogenization process in which the names of authors and institutions were checked to ensure that they were standardized to avoid subsequent errors of duplication when entering them into the various programs used.

Once this step was completed, the analysis was carried out in two steps: (1) calculation of basic bibliometric indicators, and (2) extraction of co-authorships, co-citations and the semantic map of the keywords contained in the titles and abstracts.

## 3 Results

### 3.1 Basic bibliometric indicators

**Figure 1:**
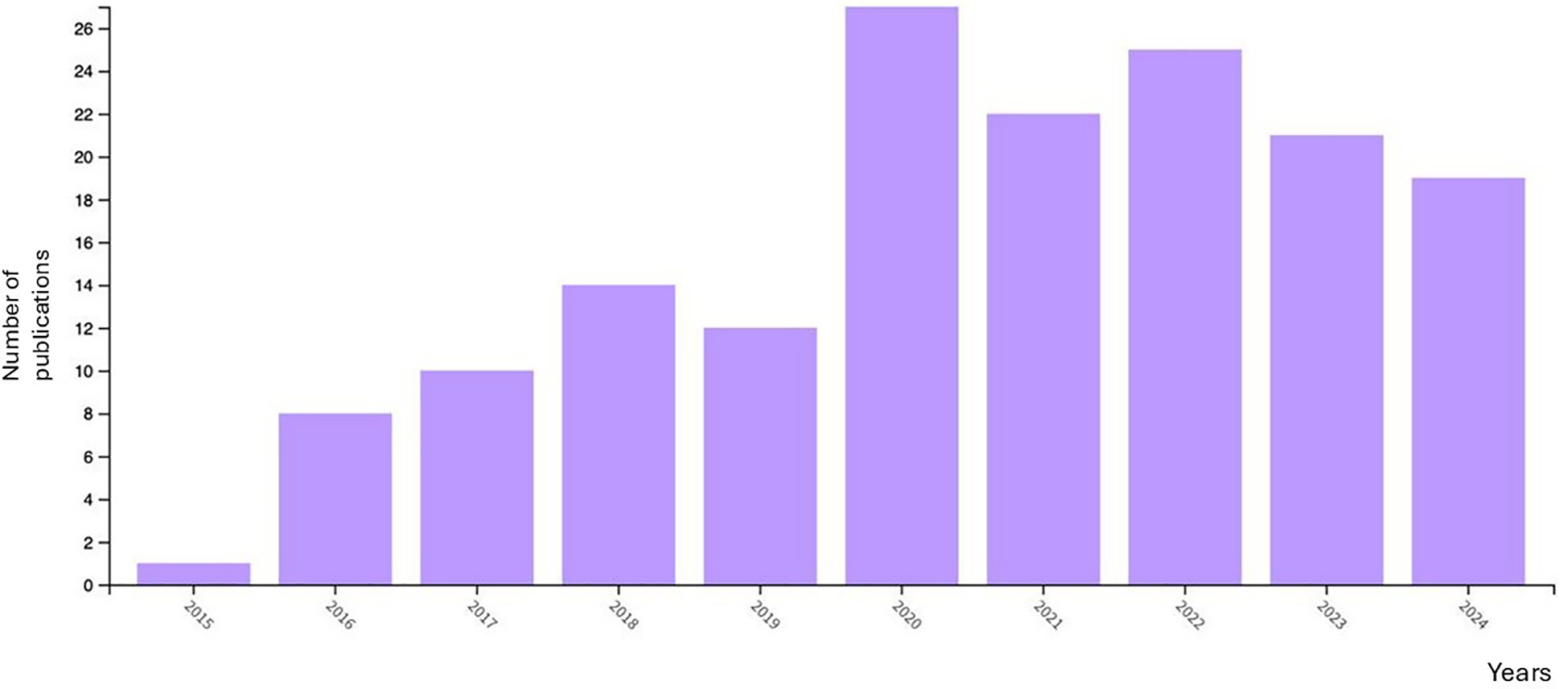
Number of publications per year. Image extracted from Web of Science through the search performed.

First, Table 1 and Figure 1 present the annual information on publications on the topic. It shows the number of papers published each year accompanied by their respective LCS (Local Citation Score) and GCS (Global Citation Score) as derived from the ISI's Web of Science database.

**Table 1:** Bibliometric analysis according to publication year.

Publication Year	Recs	Percent	LCS	GCS
2009	1	0.6	0	174
2012	2	1.3	0	431
2016	7	4.5	15	388
2017	10	6.4	7	866
2018	14	8.9	6	651
2019	10	6.4	0	274
2020	23	14.6	16	681
2021	21	13.4	3	180
2022	26	16.6	3	177
2023	21	13.4	0	55

Although no year limit was set in the search, the beginning of the documents published about the work was in 2009. Since then, and up to the present day, the years with the highest number of publications on the topic have been, in descending order, 2022, 2020, 2021, 2023 and 2024. In 2009, only 1 article was published in this database, and almost 10 years later, in 2018, a maximum of 14 articles were published per year. Taking into account the GCS per year, 2017 received the highest number of citations (GCS = 866), followed by 2020 (GCS = 681) and 2018 (GCS = 651).

**Figure 2:**
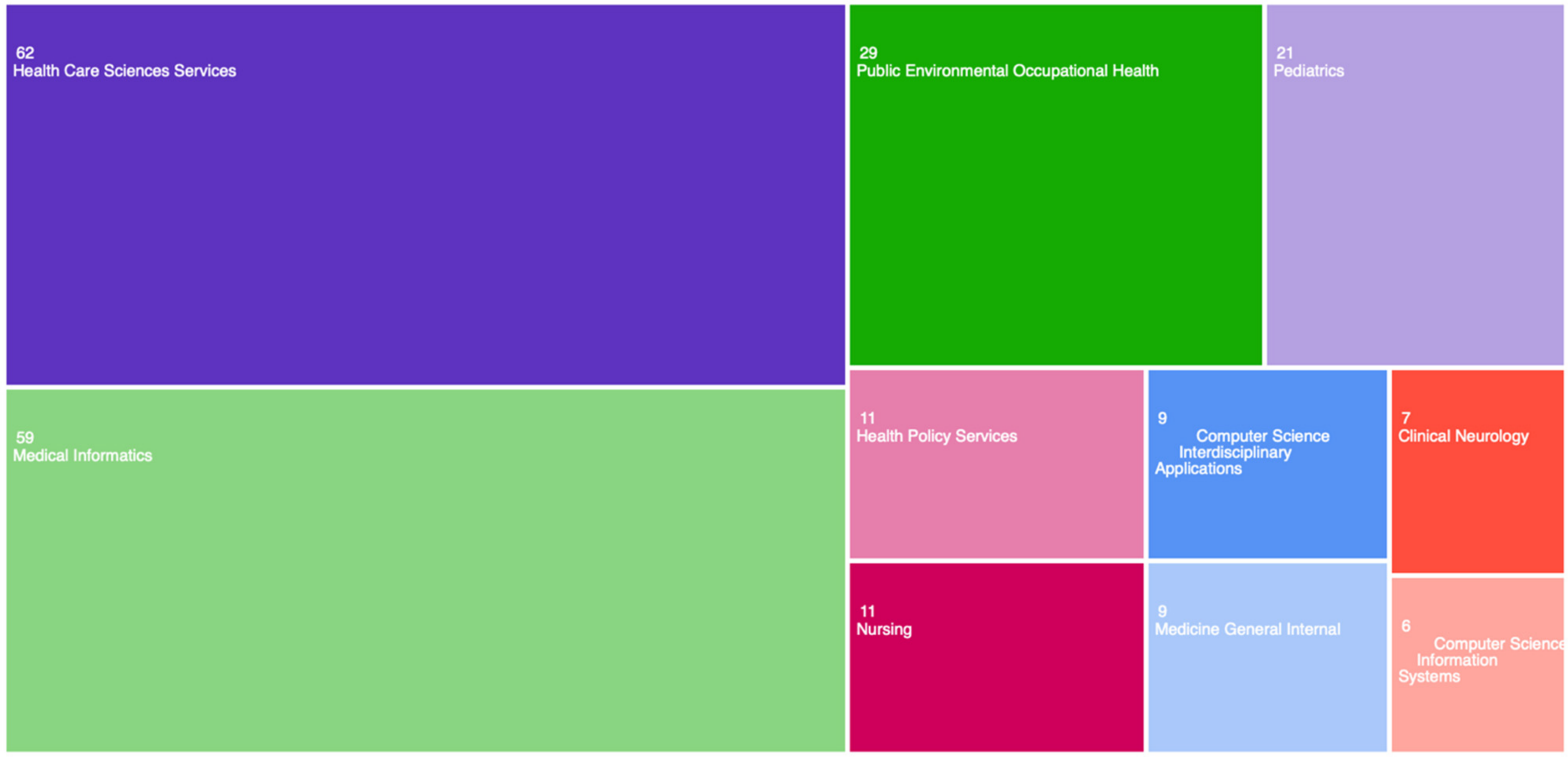
Bibliometric analysis according to research area. Image extracted from Web of Science through the search performed.

The top 10 categories in which the most articles were published within the Web of Science database are shown in Figure 2.

Table 2 below shows the top 15 scientific journals with the highest number of publications and citations related to technological interventions for chronically ill adolescents.

**Table 2:** Bibliometric analysis according to journal of publication.

Journal	Recs	Percent	LCS	GCS	LCR
Journal of Medical Internet Research	17	10.8	0	947	7
JMIR mHealth and uHealth	9	5.7	0	338	0
JMIR Formative Research	7	4.5	0	25	1
JMIR Pediatrics and Parenting	6	3.8	0	197	3
JMIR Research Protocols	5	3.2	0	69	2
Digital Health	4	2.5	0	20	2
Cochrane Database of Systematic Reviews	3	1.9	0	173	3
Games for Health Journal	3	1.9	7	106	3
International Journal of Environmental Research and Public Health	3	1.9	0	14	0
JMIR Serious Games	3	1.9	0	60	2
Journal of Pediatric Nursing	3	1.9	0	14	3
Journal of The American Medical Informatics Association	3	1.9	12	235	0
Patient Preference and Adherence	3	1.9	3	42	0
Trials	3	1.9	0	20	0
BMC Public Health	2	1.3	0	18	0

With a range between 1 and 17, the *Journal of Medical Internet Research* has a total of 17 publications and is also the most cited journal with a GCS of 947. *JMIR mHealth and uHealth,* and *JMIR Formative Research* are the next most productive journals in this area, with 9 and 7 publications respectively. The rest of the journals have six or less publications each one. On the other hand, *JMIR mHealth and uHealth* (GCS = 338) and *Journal of The American Medical Informatics Association* (GCS = 235) are the next most cited ones.

Regarding the most productive authors in the study of technological interventions for chronic diseases in adolescence, Table 3 shows the number of publications of the 10 most prominent, together with the number of times they were cited (GCS). Out of a total of 1.014 authors, only 3 published more than 5 articles, these being: Badawy S. (Northwestern University, Feinberg School of Medicine; Ann & Robert H. Lurie Children's Hospital, Division of Hematology, Oncology & Stem Cell Transplant, Chicago), Palermo T. (University of Washington, Department of Anesthesiology & Pain Medicine, Seattle; Seattle Children's Research Institute, Center of Health, Behavior & Development) and Stinson J. (Hospital for Sick Children, Child Health Evaluative Sciences, Research Institute of Toronto; University of Toronto, Lawrence Bloomberg Faculty of Nursing), with 8 publications in academic journals each, followed by de la Vega R. (Universidad de Málaga, Facultad de Psicología & Logopedia; Instituto de Investigación Biomédica de Málaga y Plataforma en Nanomedicina) and Lalloo C. (Hospital for Sick Children, Child Health Evaluative Sciences, Research Institute of Toronto; University of Toronto, Institute of Health Policy, Management & Evaluation) with 5 publications each, and Zhou C. (Seattle Children's Research Institute, Center of Child Health, Behavior & Development, Seattle; University of Washington, Department of Pediatrics) with 4 publications. On the other hand, Campbell F. (Faculty of Medical Sciences, Newcastle University), Hankins J. (St. Jude Children's Research Hospital, USA), Holtz B. (Michigan State University, Department of Advertising & Publishing Relations) and Hood K. (Standford University, Division of Pediatric Endocrinology and Diabetes, Palo Alto) have all published 3 related articles. According to the GCS value, Badawy S. is the most cited author over the years (GCS = 486), followed by Stinson J. (GCS = 164) and Hankins J. (GCS = 161).

**Table 3:** Bibliometric analysis according to authors.

Author	Recs	LCS	GCS	LCR
Badawy S	8	0	486	4
Palermo T	8	6	111	3
Stinson J	8	2	164	2
de la Vega R	5	6	64	3
Lalloo C	5	2	58	2
Zhou C	4	6	65	0
Campbell F	3	0	33	1
Hankins J	3	0	161	1
Holtz B	3	7	67	1
Hood K	3	2	108	0

As shown in Table 4, the United States was the country of origin of most of the outstanding articles in this field, with 81 publications, followed from far by the United Kingdom (21), Australia (16), Canada (16), Denmark (8), Germany (8), Spain (7), the Netherlands (6), Sweden (6) and Italy (5).

**Table 4:** Bibliometric analysis according to country.

Country	Recs	Percent	LCS	GCS
USA	81	51.6	30	2,208
UK	21	13.4	2	395
Australia	16	10.2	0	529
Canada	16	10.2	2	956
Denmark	8	5.1	8	235
Germany	8	5.1	2	221
Spain	7	4.5	0	24
Netherlands	6	3.8	3	131
Sweden	6	3.8	10	167
Italy	5	3.2	0	101

**Figure 3:**
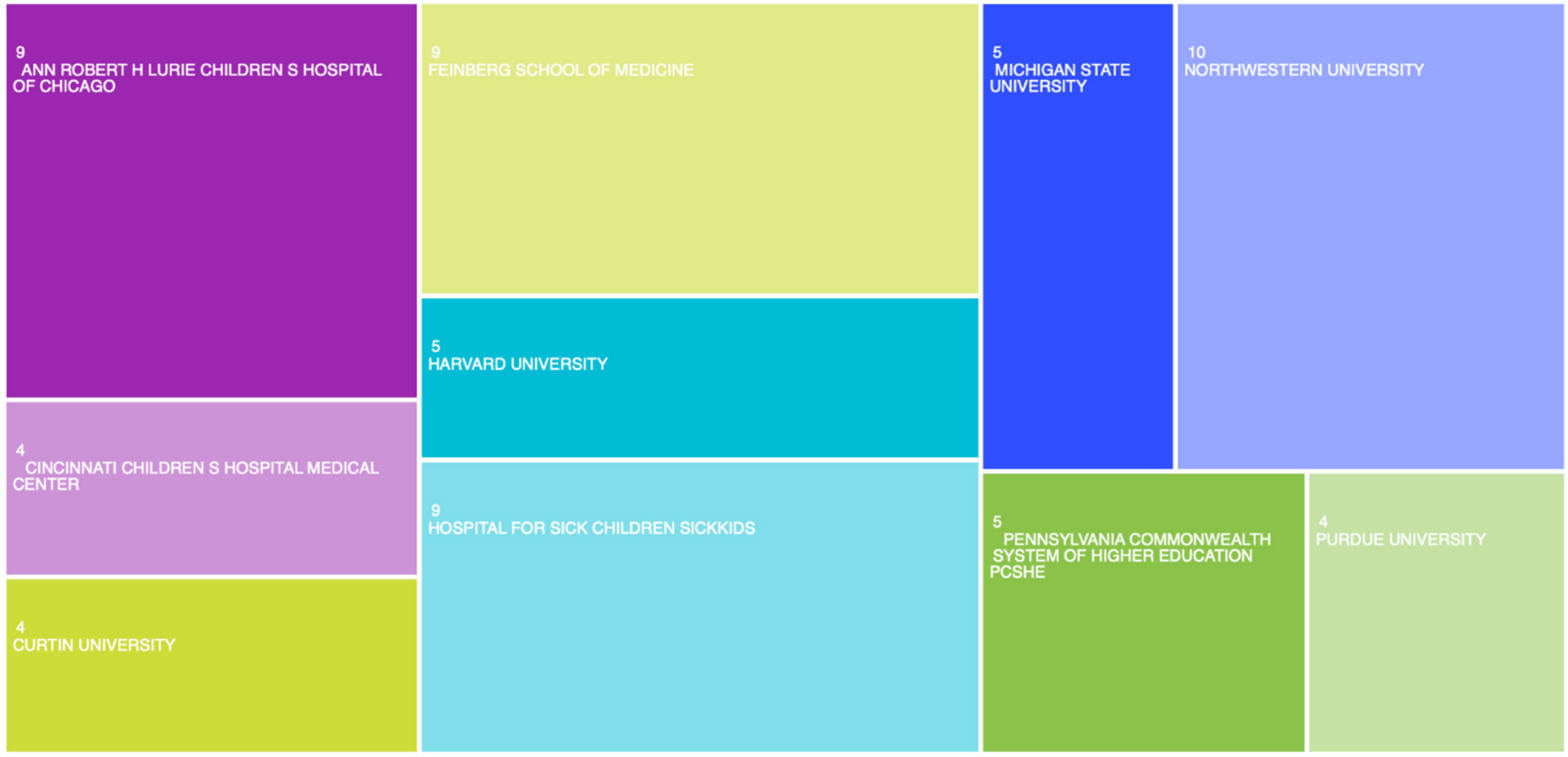
Main publishing institutions of the subject of study. Image extracted from Web of Science through the search performed.

In terms of institutions, Table 5 and Figure 3 show the 10 most prominent ones in the study on the use of new technologies in adolescents with chronic diseases. A total of 657 institutions published on this topic in WoS, with only 7 having more than 5 publications. Northwestern University had the highest volume of publications (10), followed by the Hospital for Sick Children (9), University of Washington (9), Ann & Robert H. Lurie Children's Hospital of Chicago (8), Seattle Children's Research Institute (8), University of Toronto (8), University of Sydney (7), Stanford University (5), University of Auckland (5) and University of Pittsburgh (4).

**Table 5:** Bibliometric analysis according to institution.

Institution	Recs	Percent	LCS	GCS
Northwestern University	10	6.4	5	588
Hospital for Sick Children	9	5.7	2	592
University of Washington	9	5.7	7	295
Ann & Robert H. Lurie Children's Hospital of Chicago	8	5.1	4	578
Seattle Children's Research Institute	8	5.1	6	248
University of Toronto	8	5.1	2	637
University of Sydney	7	4.5	0	73
Stanford University	5	3.2	2	121
University of Auckland	5	3.2	2	127
University of Pittsburgh	5	3.2	0	202

Table 6 shows the most important subdivisions in terms of publications in this field, together with the institution to which they belong. In this regard, the most productive subdivisions in the field were the Center for Child Health, Behavior & Development from the Seattle Children's Research Institute (*n* = 7), and the Division of Hematology, Oncology & Stem Cell Transplantation from the Ann & Robert H. Lurie Children's Hospital of Chicago (*n* = 6). The rest of subdivisions have five or less publications related each.

**Table 6:** Bibliometric analysis according to institution.

Institution with Subdivision	Recs	Percent	LCS	GCS
Seattle Children's Research Institute, Center for Child Health, Behavior & Development	7	4.5	6	97
Ann & Robert H. Lurie Children's Hospital of Chicago, Division of Hematology, Oncology & Stem Cell Transplantation	6	3.8	4	419
Northwestern University, Department of Pediatrics	5	3.2	4	419
University of Washington, Department of Anesthesiology & Pain Medicine	5	3.2	6	61
Northwestern University, Feinberg School of Medicine	4	2.5	1	163
University of Toronto, Institute of Health Policy, Management & Evaluation	4	2.5	2	38
University of Washington, Department of Pediatrics	4	2.5	2	119
Hospital for Sick Children, Child Health Evaluative Sciences	4	2.5	6	211
Hospital for Sick Children, Child Health Evaluative Sciences	3	1.9	2	32
Hospital for Sick Children, Toronto	3	1.9	0	106

Finally, the most cited articles are listed in Table 7. Following the GCS score, the study presented by Cafazzo et al. (64) was the one that received the highest volume of citations (GCS = 428), in which they present a pilot study of the design of an mHealth application focused on the self-management of the diabetes type 1 on adolescents, published in the *Journal of Medical Internet Research*. The second article that had been widely cited (GCS = 205) was the systematic review by Kitsiou et al. (65) on the effectiveness of mHealth interventions for patients with diabetes, published in the journal *PLOS ONE*. Lastly, Boushey et al. (66), published the third most cited paper (GCS = 174) in the *European Journal of Clinical Nutrition*, whose content revolves around the use of technology in children's dietary assessment.

**Table 7:** Ten most cited articles of the sample.

CITED ARTICLES	GCS
Cafazzo, J. A., Casselman, M., Hamming, N., Katzman, D. K., & Palmert, M. R. (2012). Design of an mHealth app for the self-management of adolescent type 1 diabetes: a pilot study. *Journal of medical Internet research*, *14*(3), e70. https://doi.org/10.2196/jmir.2058	428
Kitsiou, S., Paré, G., Jaana, M., & Gerber, B. (2017). Effectiveness of mHealth interventions for patients with diabetes: An overview of systematic reviews. *PloS one*, *12* (3), e0173160. https://doi.org/10.1371/journal.pone.0173160	205
Boushey, C. J., Kerr, D. A., Wright, J., Lutes, K. D., Ebert, D. S., & Delp, E. J. (2009). Use of technology in children's dietary assessment. *European journal of clinical nutrition*, *63 Suppl 1*(Suppl 1), S50–S57. https://doi.org/10.1038/ejcn.2008.65	174
Badawy, S. M., Barrera, L., Sinno, M. G., Kaviany, S., O'Dwyer, L. C., & Kuhns, L. M. (2017). Text Messaging and Mobile Phone Apps as Interventions to Improve Adherence in Adolescents With Chronic Health Conditions: A Systematic Review. *JMIR mHealth and uHealth*, *5* (5), e66. https://doi.org/i: 10.2196/mhealth.7798	153
Mendoza, J. A., Baker, K. S., Moreno, M. A., Whitlock, K., Abbey-Lambertz, M., Waite, A., Colburn, T., & Chow, E. J. (2017). A Fitbit and Facebook mHealth intervention for promoting physical activity among adolescent and young adult childhood cancer survivors: A pilot study. *Pediatric blood & cancer*, *64* (12), 10.1002/pbc.26660. https://doi.org/10.1002/pbc.26660	151
Badawy, S. M., & Radovic, A. (2020). Digital Approaches to Remote Pediatric Health Care Delivery During the COVID-19 Pandemic: Existing Evidence and a Call for Further Research. *JMIR pediatrics and parenting*, *3* (1), e20049. https://doi.org/10.2196/20049	145
Charlier, N., Zupancic, N., Fieuws, S., Denhaerynck, K., Zaman, B., & Moons, P. (2016). Serious games for improving knowledge and self-management in young people with chronic conditions: a systematic review and meta-analysis. *Journal of the American Medical Informatics Association: JAMIA*, *23* (1), 230–239. https://doi.org/10.1093/jamia/ocv100	123
Badawy, S. M., Cronin, R. M., Hankins, J., Crosby, L., DeBaun, M., Thompson, A. A., & Shah, N. (2018). Patient-Centered eHealth Interventions for Children, Adolescents, and Adults With Sickle Cell Disease: Systematic Review. *Journal of medical Internet research*, *20* (7), e10940. https://doi.org/10.2196/10940	105
Wang, X., Shu, W., Du, J., Du, M., Wang, P., Xue, M., Zheng, H., Jiang, Y., Yin, S., Liang, D., Wang, R., & Hou, L. (2019). Mobile health in the management of type 1 diabetes: a systematic review and meta-analysis. *BMC endocrine disorders*, *19* (1), 21. https://doi.org/10.1186/s12902-019-0347-6	104
Ramsey, W. A., Heidelberg, R. E., Gilbert, A. M., Heneghan, M. B., Badawy, S. M., & Alberts, N. M. (2020). eHealth and mHealth interventions in pediatric cancer: A systematic review of interventions across the cancer continuum. *Psycho-oncology*, *29* (1), 17–37. https://doi.org/10.1002/pon.5280	96

### 3.2 Co-author, co-citation and co-occurrence analysis

#### 3.2.1 Co-author

Co-authorship networks refer to the association that exists between two or more researchers when they are co-authors of one or more published articles. To present this information in a structured and comprehensible way, a graphical map of the association networks has been created, where the nodes represent the researchers and are connected if the authors to which they belong have co-authored at least one publication. To obtain this map, a minimum threshold of 2 or more co-authored papers was set.

**Figure 4:**
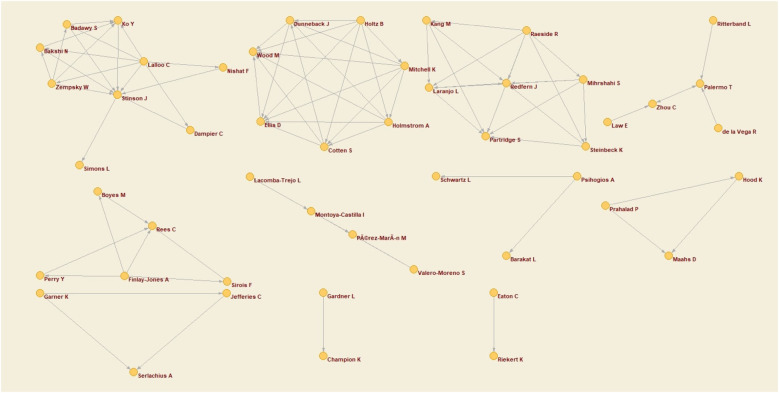
Co-author network.

As can be seen in Figure 4, the graphical map of the networks shows a total of 48 authors, organized into 11 different groups. Of these groups, the largest group has 9 members, including Badawy S. y Stinson J.—the first and third most cited author, respectively—in addition to Lalloo C.—also in the top ten most cited authors –, Ko Y., Bakshi N., Zempsky W., Nishat F., Dampier C., and Simons L. The outcome of the analysis reveals a fragmentation of the nodes into 11 distinct clusters. Notably, six of these clusters are constituted by less than five authors, suggesting a lack of consensus among researchers about the study. This fragmentation could be attributed to the limited popularity of the subject, resulting in a sparse network of authors who consistently publish articles related to it.

#### 3.2.2 Co-citation

The objective of the co-citation analysis was to ascertain the frequency with which two authors, documents, or sources are cited in tandem within a single publication. This methodological approach facilitated the identification of interconnections between these elements, thereby illuminating the intellectual underpinnings of the field of health-related quality of life within this specific population.

**Figure 5:**
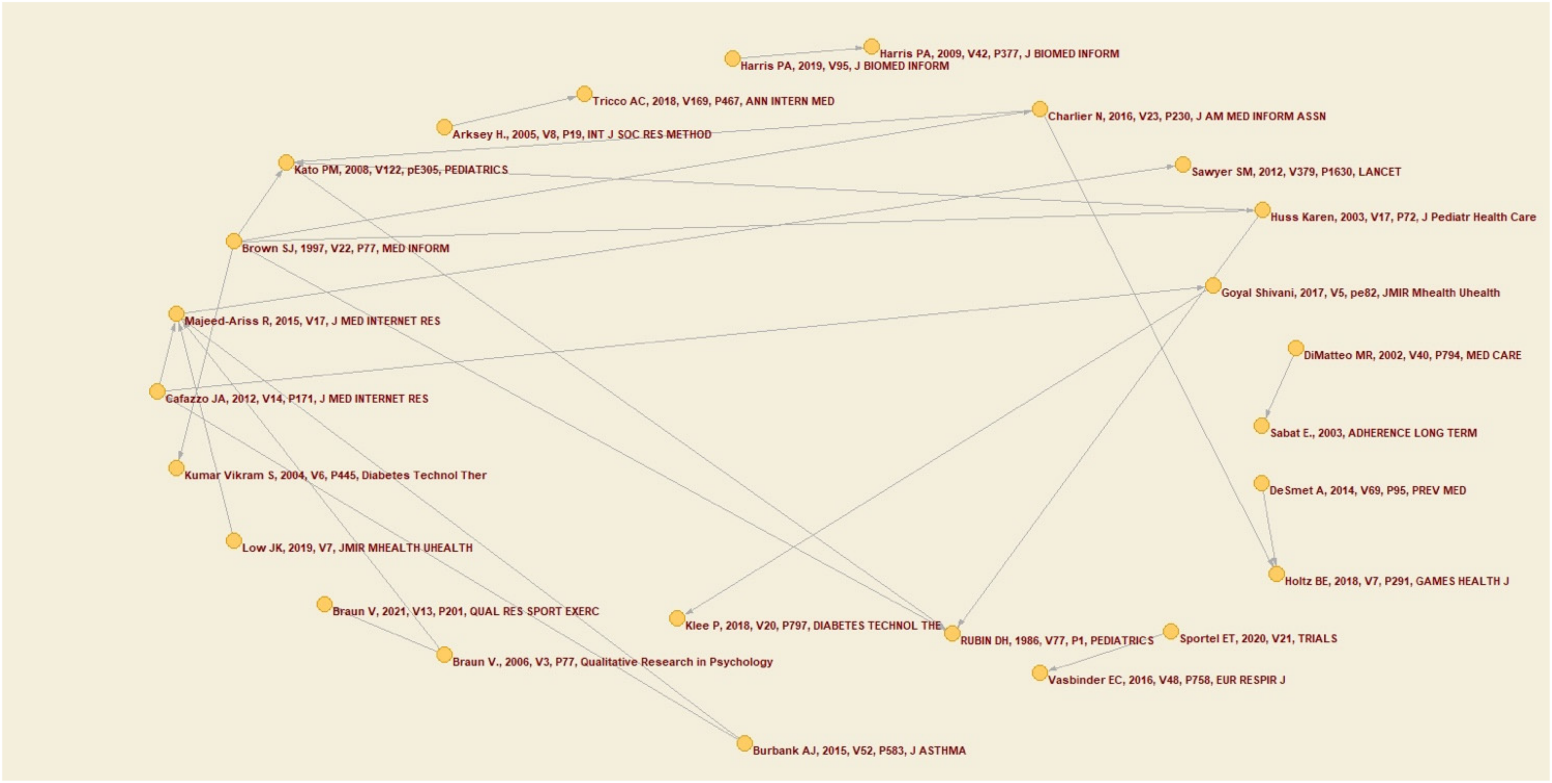
Co-citation network.

The analysis aspired to identify thematic clusters, trace the evolution of concepts and theories, and delineate the intellectual networks within the research domain. These relationships are commonly visualized through network graphs, where nodes represent authors or sources, and the links between them indicate the frequency of their co-citation. To obtain the graphical map of co-citation networks, a minimum value of 3 co-citations was set. The author with the highest number of co-citations was Rabiya Majeed-Ariss (2015), followed by Pamela M. Kato (2008), David H. Rubin (1986) and Bree Holtz (2018). Figure 5 shows this information graphically. These results are particularly noteworthy given that these authors do not appear among the most cited of the sample extracted, according to their GCS.

#### 3.2.3 Co-occurrence

**Figure 6:**
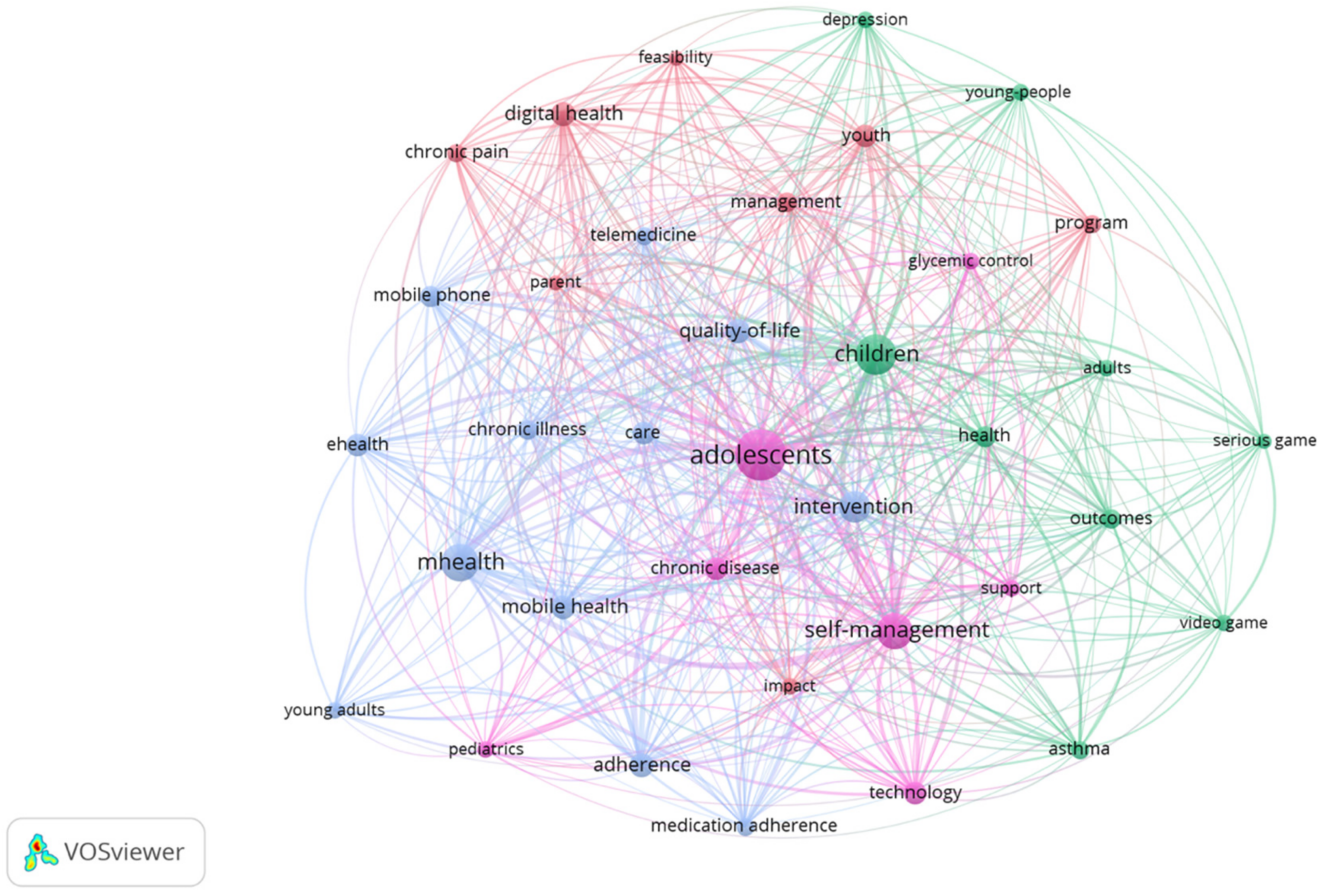
Groups of thematic analysis. Image extracted from Vosviewer through the search performed.

To try to group the different keywords used in the titles and abstracts of the analyzed articles within a specific topic, a network map was created, as shown in Figure 6. To obtain this graphic, the program VOSviewer was used, and a binary count was made, with a frequency of co-occurrence of at least 10, showing 38 items. Of these, those related to the methodology of the published study were excluded, resulting in a total of 36 terms in 4 clusters.

The concepts with the highest number of citations were “adolescents”, “children”, “self-management”, and “mHealth”. The 3 clusters around which the keywords have been grouped are highlighted in different colors. In blue we would find terms related to health and well-being in adolescents and young adults, including *adolescents, children, youth, young people, quality of life, management, glycemic control, depression e impact*. In green would be all the words related to digital technologies and mobile health in chronic diseases, such as mHealth*, eHealth, mobile health, telemedicine, digital health, mobile phone, pediatrics, young adults, chronic illness, chronic pain*. Finally, the third cluster, shown in pink, relates to self-management and adherence in chronic disease, including concepts such as *self-management, adherence, medication adherence, asthma, chronic disease, intervention, support, serious game, program, outcomes y technology*.

**Figure 7:**
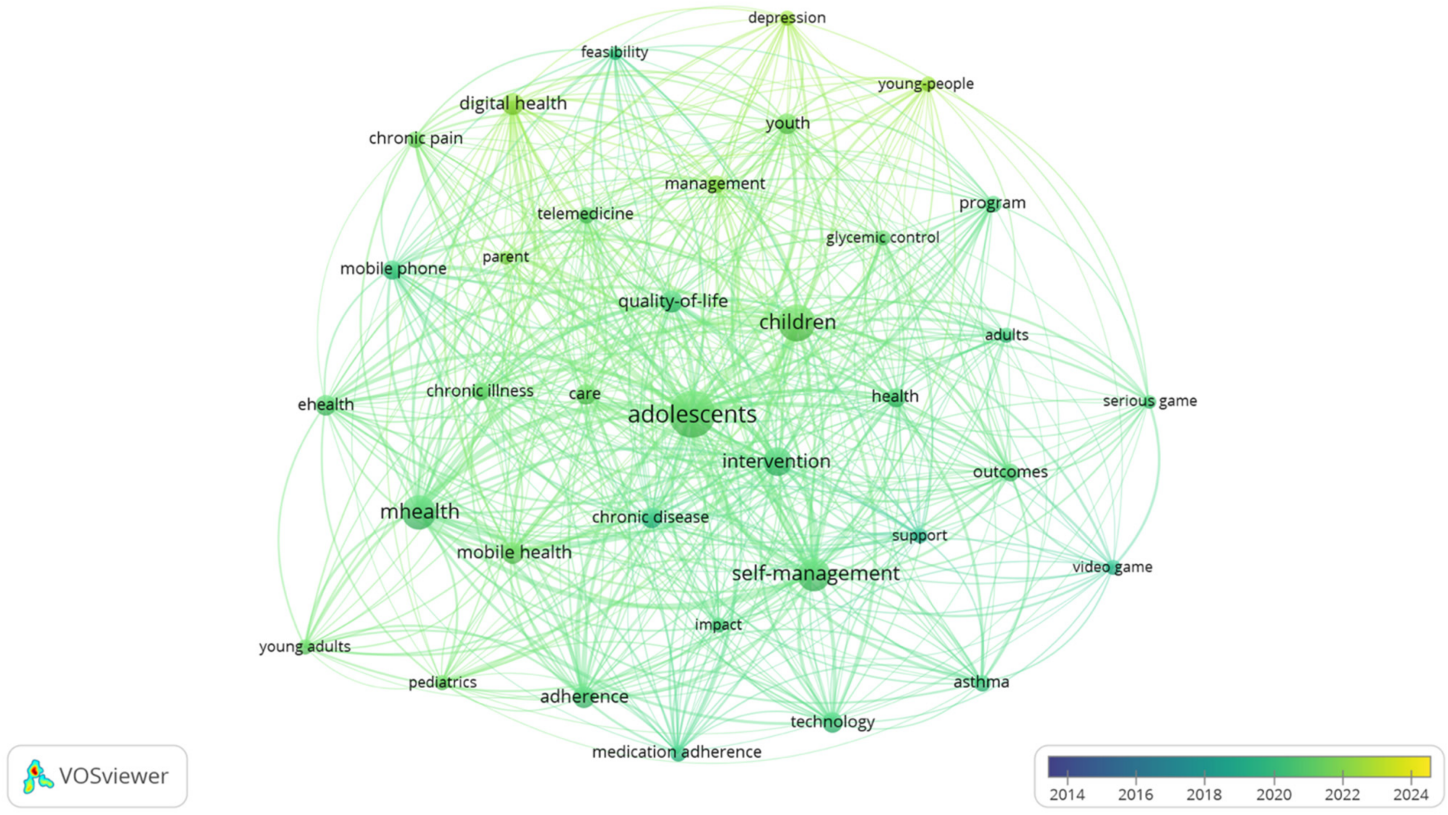
Co-occurrence network: progression of keywords per year. Image extracted from Vosviewer through the search performed.

Figure 7 also shows the density of use of these terms over the last few years, with the subject matter of the different articles published remaining generally constant. A close examination of the temporal patterns in appearance and disappearance of keywords in literature reveals no significant fluctuations in their frequency. This observation suggests that the field of study has remained relatively stable, devoid of any substantial innovations or paradigm shifts over the examined period. Nevertheless, there is a discernible inclination towards investigating the evolution of digital health applications designed to facilitate self-management and to address depressive symptomatology associated with chronic diseases in young people.

**Figure 8:**
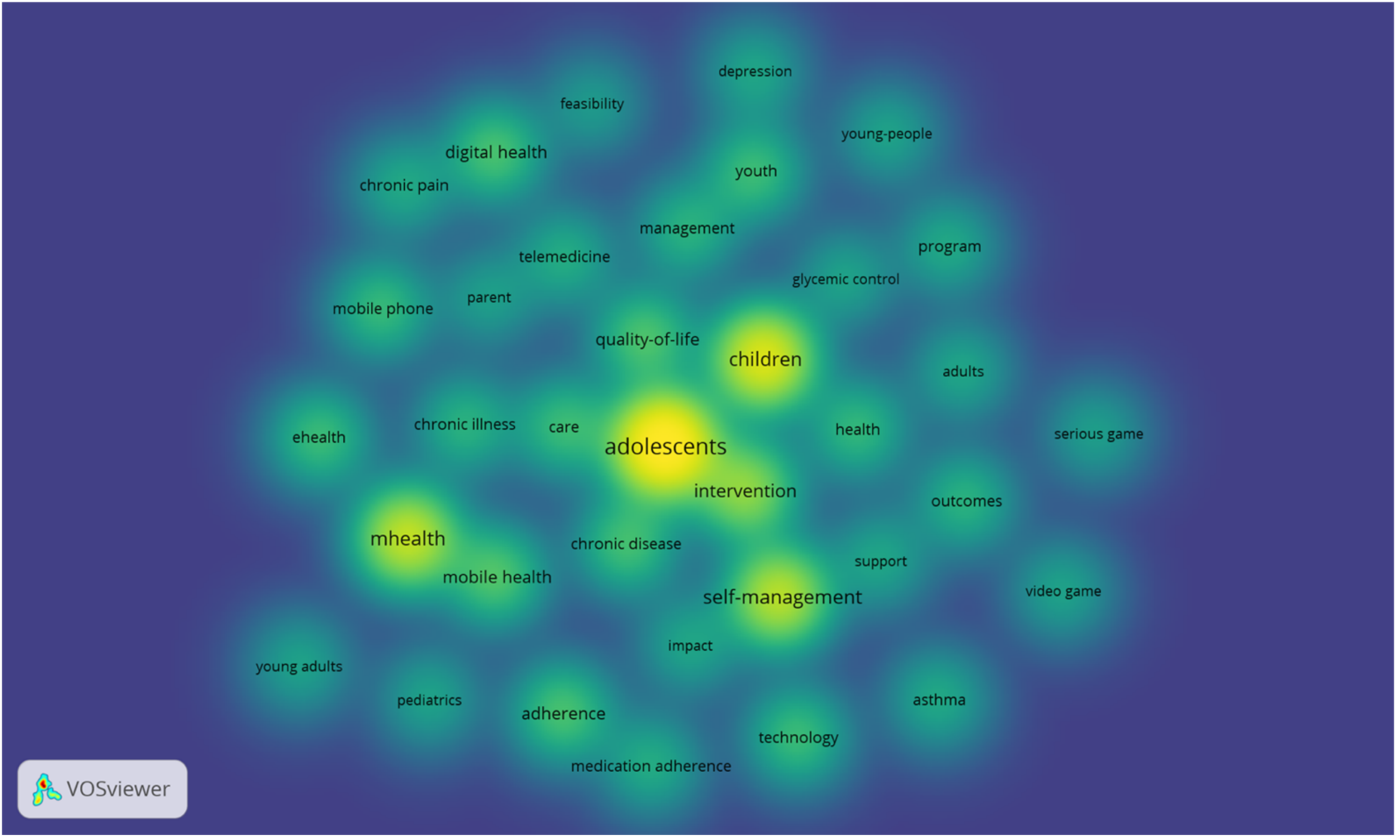
Co-occurrence network: density visualization. Image extracted from Vosviewer through the search performed.

Lastly, Figure 8 shows a map of the density of the same terms as above, represented by different intensities of each color. In this sense, the higher the intensity, the greater the number of times the word was used in the titles and abstracts of the articles, with yellow being associated with higher densities, green with moderate densities and blue with lower densities. As previously stated, the terms “adolescents,” “children,” “mHealth,” and “self-management” exhibit the highest density.

## 4 Discussion

Chronic diseases represent a significant healthcare challenge (67), exerting considerable influence on the various domains of patients' lives (11, 68). Particularly during adolescence, the presence of a chronic condition can act as an additional stressor, compounding the challenges inherent to this developmental stage (69, 70). Appropriate management and coping strategies are imperative to address and prevent current and future problems, thereby enhancing the quality of life for these individuals (71, 72). To this end, to promote and improve the overall health of these patients, it is essential to develop interventions focused on the prevention and treatment of these conditions. While the conventional intervention format has demonstrated efficacy in various domains related to chronic illness, there are still some limitations that hinder access for diverse populations (73, 74). Digital interventions, such as mHealth, have been shown to address some of these limitations, demonstrating their efficacy in enhancing the management of chronic diseases, including diabetes (46, 47) or cystic fibrosis (50).

Some bibliometric studies have been published about chronic diseases, investigating various aspects, including the management of chronic diseases within the context of relationships (75), the perception of illness (76) or the application of Artificial Intelligence (AI) in the context of chronic diseases (77). However, none of these studies specifically focus on the use of digital interventions or mHealth in chronic diseases during adolescence. Since this stage is of paramount importance in the developmental process towards adulthood, it is imperative for health professionals to concentrate on the potential issues that may emerge during this period, particularly in cases where chronic diseases are present.

Despite the utilization of novel technologies, and the term “mHealth”, dating back to earlier periods, it was not until 2009 that the inaugural article was published in WoS, associating one of these concepts with the prevalence of chronic diseases in adolescence. After this, there has been a moderately upward trend in the number of publications over the years, with this increase becoming more evident following the advent of the pandemic COVID-19. This development has necessitated a global response, entailing adaptation to a series of novel challenges and shifts in healthcare delivery (10, 78). The digital realm has emerged as a pivotal platform, offering the potential for both patient and professional care, while ensuring the continuity of essential healthcare services. The mounting demand within the sector gave rise to a pressing need for a digital update to address these emerging imperatives, thus prompting a notable surge in research interest and a substantial increase in scientific publications related to the topic (79). Following the passage of time and the return to relative normality, interest in these interventions may have declined, although the overall data show a clear upward trend.

Extant scientific literature supports the existence of a direct relationship between the presence of psychological and emotional problems and poorer adherence to medical treatment, which can have negative implications for the physical health of these patients. However, as can be seen in the results, the main categories in which the works published in this field fall into belong to the medical or technological field, with very little presence in the psychological field. Despite ongoing exploration of technological resources to enhance interventions for pediatric populations with chronic illnesses, these endeavors predominantly focus on physical health aspects, frequently overlooking the significance and impact of the psychosocial domain.

Contrary to the focus of other studies on the clinical effectiveness of digital interventions or on the technological development of these tools, the present study examines the evolution of scientific knowledge in this field, identifying trends, gaps and future opportunities through bibliometric analysis. This analysis helps to clarify, understand and update the current state and evolutionary trend in relation to scientific research on the development and implementation of new technologies when intervening in aspects related to the management of chronic disease during adolescence. The paucity of studies that bring these concepts together is thus evident, along with the need to continue to advocate for this line of research to contribute to the generation of a solid scientific basis from which to develop new, more accessible interventions that improve the quality of life of adolescents with chronic disease. The increased survival of these patients, in conjunction with the growing prevalence of chronic diseases, underscores the necessity to comprehend and consider all factors that may influence the overall well-being of the individual and their quality of life. Facilitating access to interventions or prevention programs by different health professionals has the potential to engender significant improvements for these adolescents, both at the physical and psychosocial-emotional levels, which are subsequently reflected in adulthood. Methodologically, this study not only analyses scientific production in terms of volume of publications, but also examines patterns of collaboration between authors, thematic distribution through keywords and temporal evolution of research in this area. This comprehensive approach enables us to comprehend the current state of knowledge in this field and to identify emergent trends that can guide future research. In this sense, the information presented here on the most cited journals, most used keywords and most relevant authors within the field of chronic disease and digital interventions reveals the future development trends within this line of research, thus guiding researchers and health professionals towards key aspects to focus on in future work. In this way, it contributes to the improvement and relevance of scientific research within this sector. The findings of this analysis are of pertinence in the post-pandemic context, wherein the digitization of healthcare has been elevated to a primary concern. The present study underscores the way the pandemic precipitated the adoption of digital technologies in the management of chronic diseases among adolescents. However, it is also evident that this growth has not been uniform across all domains of healthcare.

However, it is important to acknowledge the limitations of this work, which are crucial to consider for future research in this field. Firstly, the search for articles was confined to the Web of Science database and its main collection, potentially resulting in the exclusion of some papers from other databases. Additionally, while the field of chronic illness has seen a significant amount of research, comparable to other areas, the implementation of digital applications or programs to intervene in these conditions remains limited. This scarcity is particularly pronounced when focusing on the adolescent stage, a period marked by heightened vulnerability to change. Consequently, the number of articles included in this study for bibliographical analysis is notably reduced. Finally, despite the relevance of the information regarding the evolution trends and current state of research in this field, it should be noted that the content of the included articles has not been analyzed in its entirety, and therefore some may have been accepted if they did not provide relevant information in relation to the subject matter.

This bibliometric analysis provides a comprehensive overview of the current state of research on digital interventions for chronically ill adolescents. It also highlights critical areas that require further attention, including the identification of gaps in the integration of psychosocial aspects within these interventions. These findings underscore the necessity for more holistic and multidisciplinary approaches in research. As the digitization of healthcare continues to evolve, these findings can serve as a roadmap to guide future research and develop more effective, accessible and patient-centered strategies. Consequently, this study contributes to the consolidation of knowledge in a developing field, advocating a more balanced and holistic approach to the design of interventions for this vulnerable population.
